# Comparison of Barriers Against Mammography Screening in Socioeconomically Very Low and Very High Populations

**DOI:** 10.7759/cureus.690

**Published:** 2016-07-14

**Authors:** Tolga Ozmen, Atilla Soran, Vahit Ozmen

**Affiliations:** 1 General Surgery, Marmara University Hospital; 2 Comprehensive Breast Centre, Magee Womens Hospital of UPMC; 3 Department of Surgery, Istanbul University

**Keywords:** breast cancer, socioeconomical status, mammography screening, prevention, population based, cross sectional

## Abstract

**Aim:**

To compare barriers against mammography screening (MS) in socioeconomically the most and the least developed cities in a developing country, Turkey.

**Methods:**

We compared two population-based survey studies investigating barriers against MS in women aged 40-69 years and living in the lowest socioeconomic status (SES) city (Mus) and the highest SES city (Istanbul/Bahcesehir).

**Results:**

In Mus 2,054 and in Bahcesehir 908 women were surveyed. MS rate was higher in Bahcesehir (49% vs. 35%, p<0.001). Being younger than 50 years old and having no insurance were barriers against MS in Mus. Being older than 60 years of age, widowhood, being illiterate, unemployment, a monthly income lower than the hunger threshold and limited insurance coverage were barriers against MS in Bahcesehir. Both in Mus and in Bahcesehir women not reading the daily newspaper and not making annual gynecology visits had lower MS rates. Both in Mus and in Bahcesehir audiovisual media was the most common source of information about breast cancer (BC). Women, who had a chance to be informed about BC by their doctors, had a higher MS rate. Being knowledgeable about BC being the most common cancer in females increased MS rates in both cities, while a false belief of MS exposing to unnecessary radiation decreased MS rates in both cities.

**Conclusion:**

Both in high and low SES populations more efforts should be given to influence women with low educational level, low-income level, and having no/limited insurance, while designing BC awareness programs. Low SES populations women being in 40-49 years age interval, in high SES populations being in 60-69 years age interval, and widowhood were SES-specific barriers and should be taken into consideration. Audiovisual media should be used efficiently to educate women on BC. Physicians from all specialties should not miss the chance to refer their patients to MS.

## Introduction

Breast cancer (BC) is the most common cancer and the second cause of cancer-related mortality in women [1-2]. According to the literature, BC mortality is 40% reduced by inviting women older than 40 years of age to mammography screening (MS) [3]. Nevertheless, screening rate remains low in developing and underdeveloped countries [4-5].

Studies showed that health beliefs of the population play a major role in health practices. If the population is better known, models can be adjusted for that population and successful outcomes can be achieved [1, 6-7].

Turkey is a developing country with socioeconomically heterogeneous populations living in different regions of the country, and according to socioeconomic parameters, eastern regions of Turkey have always been the lowest of all other regions and western regions the highest [8]. In July 2004, BC screening guidelines were issued, and screening centers have been organized by the Ministry of Health in Turkey. Despite these efforts, screening is still performed primarily on an opportunistic basis rather than on an organized basis with a small fraction of women undergoing screening.

Herein we aimed to compare the barriers against MS between two socioeconomically contrarious populations. We also wanted to analyze discrepancies in health behaviors and general health motivation between these two different populations. We believe the results of our study will be useful in understanding breast health practices in socioeconomically different populations and implementing customized MS programs in different population types.

## Materials and methods

This is a study comparing the results of two population-based, cross-sectional survey studies, which used the same questionnaire to determine barriers against MS. According to the Ministry of Development, Mus (a city in Eastern Turkey) and Istanbul are socioeconomically the lowest and the highest status cities in Turkey, respectively [9]. Bahcesehir is a county in Istanbul where a more homogenous population with a high socioeconomic status is living. Both studies used the 'cluster sampling method' as their random sampling method. Women aged 40 to 69 years and living in Mus and in Bahcesehir formed the two sampling units of these two studies. Participants who agreed to participate were explained the nature and the objectives of the study, and informed consent was formally obtained. No reference to the participants' personal identities were made at any stage during data analysis or in the paper.

The interviewers asked questions, which included three main topics: descriptive information, breast health history, and BC awareness. Under the BC awareness topic, the main source for information on BC, the knowledge on mammography (MG) being free of cost, BC being the most common cancer in females, BC being curable if diagnosed early, and whether MG exposing women to unnecessary radiation questions were asked. Women, who had a screening MG in the last two years, were accepted as screened in both studies. The answers were compared between two studies.

Student’s t‐test was used for continuous variables, and chi‐square test was used for categorical variables. Statistical package for social sciences (SPSS) software (version 20.0) was used for analysis. P values of less than 0.05 were considered as statistically significant.

## Results

In Mus 2,054 women were surveyed while in Bahcesehir 908 women were surveyed. The participation rates were 89.1% in Bahcesehir and 85% in Mus. Mean age was similar both in Mus and in Bahcesehir (49.5 ±8.3{40-69}, 49.6 ±7.4{40-69}, respectively; p= 0.777). The marital status differed between the two populations with the rate of marriage being 88% (n=1724) in Mus and 43% (n=93) in Bahcesehir (p<0.001). The literacy rate was 95% in Bahcesehir and 44% in Mus (p<0.001). The rate of women graduated from at least high school was 77% in Bahcesehir and 14% in Mus (p<0.001). The proportion of women daily reading a newspaper was 73% in Bahcesehir and 9% in Mus (p<0.001), (Table 1).  

Rates of working and retired women were 22% and 25% respectively in Bahcesehir and 5% and 2% respectively in Mus (p<0.001). The mean monthly income was $1,942 ±1280{148-4926} in Bahcesehir and $687 ±445 {0-4187} in Mus (p<0.001). In Bahcesehir the rate of having a social security insurance was 86%, and rate of having a Green Card (a non-contributory health insurance program in Turkey for the poor and without formal social insurance coverage) was 1%; while in Mus these rates were 66% and 24%, respectively (p<0.001). In Bahcesehir 56% of the women were visiting a gynecologist annually, while in Mus this rate was 15% (p<0.001). MS rate was 35% in Mus and 49% in Bahcesehir, (p<0.001), (Table 1).

**Table 1:** Comparison of characteristics of two populations Categorical data are presented as n (%). Continuous data are presented as mean ±SD {range}. **Green Card, non-contributory health insurance program in Turkey for the poor and without formal social insurance coverage.

	Mus	Bahcesehir	P
Age (y)	49.5 ±8.3 {40-69}	49.6 ±7.4 {40-69}	0.777
Marital Status			
Married	1724 (88)	93 (43)	
Single	28 (1)	63 (30)	<0.001
Widowed	210 (11)	57 (27)	
Literacy	865 (44)	861 (95)	<0.001
Graduation			
None	1213 (59)	47 (5)	
Elementary	562 (27)	165 (18)	<0.001
Middle/high school	232 (11)	415 (46)	
University	49 (3)	281 (31)	
Reading ≥1 newspaper a day	161 (9)	659 (73)	<0.001
Working status			
Working	87 (5)	200 (22)	
Retired	46 (2)	228 (25)	<0.001
Non-working	1817 (93)	480 (53)	
Insurance status			
Social security	1269 (66)	779 (86)	
Green Card^**^	475 (24)	1 (1)	<0.001
Not insured	128 (7)	65 (7)	
Private insurance	59 (3)	60 (6)	
Monthly income ($)	687 ±445 {0-4187}	1942 ±1280 {148-4926}	<0.001
Annual gynecology visit	291 (15)	504 (56)	<0.001
Having MG in last 2 years	678 (35)	448 (49)	<0.001

### Comparison of the effect of descriptive characteristics on MS in two populations

In Mus women in the 40-49 year age interval were less likely to participate in MS compared to the 50-59 year and 60-69 year age intervals (30%, 42%, and 36%, respectively; p<0.001). In Bahcesehir women in the 60-69 year age interval were less likely to participate in MS compared to 40-49 year and 50-59 year age intervals (37%, 48%, and 58%, respectively; p<0.001). MS rates among different marital statuses were similar in Mus (married = 35%, single = 29%, and widowed = 36%; p=0.711); while in Bahcesehir widowed women (32%) were less likely to participate to MS compared to single (48%) and married women (59%), (p<0.012), (Table 2).

In Mus literacy did not affect the likelihood of participating in MS (literate women = 34% and non-literate women = 35%; p=0.665); while in Bahcesehir non-literate women were less likely to participate in MS (23% vs. 51%, p<0.001). In Mus MS rates were similar between different graduation levels (university = 43%, high-school = 31%, middle-school = 34%, elementary school = 34%, and no graduation = 33%; p=0.605). In Bahcesehir women with no graduation had a lower MS rate (23%) compared to women who graduated from elementary school (41%), from middle-school (40%), from high-school (52%), or from university (59%), (p<0.001). Women who did not daily read a newspaper had a lower MS rate both in Mus (34% vs. 44%, p=0.007) and in Bahcesehir (40% vs. 53%, p<0.001) (Table 2).

MS rates among different working statuses were similar in Mus (working = 36%, not-working = 35%, and retired = 48%, p=0.168). In Bahcesehir the MS rate was the lowest among not-working women (45%) compared to working (54%) and retired women (56%) (p=0.01). In Mus MS rate was lowest among non-insured women (24%) compared to women with a Green Card (29%), women with a private insurance (31%), and women having social security (39%), (p<0.001). In Bahcesehir women with a Green Card (0%) had a lower MS rate compared to non-insured women (34%), women having social security (50%), and women with a private insurance (57%), (p=0.032). In Mus MS rates were similar between different monthly income levels (higher than poverty threshold = 44%, between poverty and hunger thresholds = 37%, and lower than hunger threshold = 34%; p=0.315). In Bahcesehir MS rate was lowest among women with a monthly income level lower than hunger threshold (40%) compared to women with a monthly income between poverty and hunger thresholds (44%), and women with a monthly income higher than the poverty threshold (58%), (p<0.001), (Table 2).

**Table 2:** Comparison of the effect of descriptive parameters on the MS* rate in two populations Data are presented as n (%) *MS = mammography screening **Green Card, non-contributory health insurance program in Turkey for the poor and without formal social insurance coverage. § HT, hunger threshold ($603 according to Confederation of Turkish Trade Unions; www.turkis.org.tr) ¶ PT, poverty threshold ($1966 according to Confederation of Turkish Trade Unions; www.turkis.org.tr)

			MS^*^ (+)	MS^*^ (-)	p
Age	Mus	40-49	320 (30)	749 (70)	<0.001
		50-59	253 (42)	344 (58)	
		60-69	106 (36)	187 (64)	
	Bahcesehir	40-49	244 (48)	265 (52)	<0.001
		50-59	162 (58)	118 (42)	
		60-69	42 (37)	73 (63)	
Marital status	Mus	Married	596 (35)	1128 (65)	0.711
		Single	8 (29)	20 (71)	
		Widowed	76 (36)	134 (64)	
	Bahcesehir	Married	54 (59)	38 (41)	0.012
		Single	30 (48)	33 (52)	
		Widowed	18 (32)	38 (68)	
Literacy	Mus	Yes	295 (34)	570 (66)	0.665
		No	383 (35)	710 (65)	
	Bahcesehir	Yes	437 (51)	420 (49)	<0.001
		No	11 (23)	36 (77)	
Graduation	Mus	None	396 (33)	817 (67)	0.605
		Elementary	190 (34)	372 (66)	
		Middle School	35 (34)	67 (66)	
		High School	40 (31)	90 (69)	
		University	21 (43)	28 (57)	
	Bahcesehir	None	11 (23)	36 (77)	<0.001
		Elementary	67 (41)	98 (59)	
		Middle School	31 (40)	46 (60)	
		High School	174 (52)	161 (48)	
		University	165 (59)	115 (41)	
Reading a newspaper daily	Mus	Yes	71 (44)	90 (56)	0.007
		No	577 (34)	1142 (66)	
	Bahcesehir	Yes	350 (53)	306 (47)	<0.001
		No	98 (40)	150 (60)	
Working status	Mus	Working	31 (36)	56 (64)	0.168
		Not-working	626 (35)	1191 (65)	
		Retired	22 (48)	24 (52)	
	Bahcesehir	Working	106 (54)	92 (46)	0.01
		Not-working	215 (45)	264 (55)	
		Retired	127 (56)	100 (44)	
Insurance status	Mus	None	31 (24)	97 (76)	<0.001
		Green Card^**^	136 (29)	339 (71)	
		Social Security	489 (39)	780 (61)	
		Private	18 (31)	41 (69)	
	Bahcesehir	None	22 (34)	43 (66)	0.032
		Green Card^**^	0 (0)	1 (100)	
		Social Security	390 (50)	385 (50)	
		Private	34 (57)	26 (43)	
Monthly income ($)	Mus	< HT^§^	288 (34)	549 (66)	0.315
		HT^§^-PT^¶^	243 (37)	422 (63)	
		>PT^¶^	20 (44)	25 (56)	
	Bahcesehir	< HT^§^	40 (40)	61 (60)	<0.001
		HT^§^-PT^¶^	148 (44)	191 (56)	
		>PT^¶^	215 (58)	158 (42)	

### Comparison of the effect of health history on MS in two populations

In Mus women with a normal body weight had a lower MS rate (27%) compared to women who are overweight (35%) and obese (38%), (p=0.003). In Bahcesehir MS rates were similar between women with different BMI levels (normal BMI = 52%, overweight women = 49%, and obese women = 44%; p=0.314). In Mus women with no priorly diagnosed disease were less likely to participate in MS compared to women with a priorly diagnosed disease (29% vs. 41%, respectively; p<0.001). In Bahcesehir the MS rate did not differ between women who were diagnosed with a disease and those who were not (50% and 50%, respectively; p=0.996). Making annual gynecology visits increased the MS rate both in Mus (51% vs. 32%, p<0.001) and in Bahcesehir (68% vs. 26%, p<0.001), (Table 3).

Having a breast symptom increased the MS rate both in Mus (51% vs. 28%, p<0.001) and in Bahcesehir (64% vs. 48%, p=0.002). In Mus having a family member with BC (41% vs. 34%, p=0.04) and in Bahcesehir having a friend with BC (54% vs. 45%, p=0.005) increased the MS rates (Table 3).

**Table 3:** Comparison of the effect of health history on the MS* rate in two populations Data are presented as n (%) * MS = mammography screening ** BMI = body mass index *** BC = breast cancer

			MS^*^ (+)	MS^*^ (-)	
BMI^**^	Mus	Normal	66 (27)	183 (73)	0.003
		Overweight	312 (35)	585 (65)	
		Obese	290 (38)	466 (62)	
	Bahcesehir	Normal	232 (52)	216 (48)	0.314
		Overweight	160 (49)	169 (51)	
		Obese	56 (44)	70 (56)	
Priorly diagnosed disease?	Mus	Yes	384 (41)	553 (59)	<0.001
		No	296 (29)	714 (71)	
	Bahcesehir	Yes	228 (50)	232 (50)	0.996
		No	220 (50)	224 (50)	
Annual gynecology visit?	Mus	Yes	149 (51)	142 (49)	<0.001
		No	529 (32)	1113 (68)	
	Bahcesehir	Yes	343 (68)	161 (32)	<0.001
		No	104 (26)	294 (74)	
Any breast symptoms?	Mus	Yes	239 (51)	229 (49)	<0.001
		No	442 (28)	1135 (72)	
	Bahcesehir	Yes	66 (64)	37(36)	0.002
		No	382 (48)	419 (52)	
Family history of BC^***^	Mus	Yes	106 (41)	154 (59)	0.04
		No	576 (34)	1107 (66)	
	Bahcesehir	Yes	58 (58)	42 (42)	0.073
		No	390 (49)	414 (51)	
Friend with BC^***^?	Mus	Yes	162 (39)	259 (61)	0.104
		No	520 (34)	1000 (66)	
	Bahcesehir	Yes	248 (54)	210 (46)	0.005
		No	199 (45)	245 (55)	

### Comparison of the effect of BC knowledge on MS in two populations

Although not statistically significant, television was the most common source of information on BC both in Mus (35%) and in Bahcesehir (46%). In Mus women who reported doctors as their main source of information on BC had a higher MS rate (42%) compared to women who reported TV (36%) and friends (32%) (p<0.001).  Also in Bahcesehir women who reported doctors as their main source of information on BC had a higher MS rate (67%) compared to women who reported TV (47%) and friends (44%), (p<0.001). The phrase "I would get a MG if my doctors refers me to" was agreed by the majority of populations in Mus (92%) and in Bahcesehir (93%). In Mus women who agreed to this phrase had a higher MS rate (36% vs. 26%, p=0.015). In Bahcesehir women who agreed to this phrase had a lower MS rate, but the difference was not statistically significant (49% vs. 55%, p=0.327). Having awareness of BC being the most common cancer in females increased the MS rate both in Mus (36% vs. 28%, p=0.024) and in Bahcesehir (52% vs. 44%, p=0.042). In Mus the MS rate was higher among women who agreed to the phrase "BC is curable, if detected in an early stage" (36% vs. 25%, p=0.016). Nevertheless, in Bahcesehir MS rates were similar between women who agreed to this phrase (50%) and those who did not (53%), (p=0.786). Women who believed that MG exposes them to unnecessary radiation had a lower MS rate both in Mus (32% vs. 39%, p=0.002) and in Bahcesehir (38% vs. 53%, p<0.001), (Table 4).

**Table 4:** Comparison of the effect of BC* knowledge on the MS** rate in two populations Data are presented as n (%) * BC = breast cancer ** MS = mammography screening *** MG= mammography

			MS^**^ (+)	MS^**^ (-)	p
Source of info on BC^*^	Mus	TV (35%)	303 (36)	545 (64)	<0.001
		Doctors (31%)	313 (42)	442 (58)	
		Friends (34%)	259 (32)	542 (68)	
	Bahcesehir	TV (46%)	250 (47)	283 (53)	<0.001
		Doctors (29%)	226 (67)	110 (33)	
		Friends (25%)	124 (44)	161 (56)	
I would get MG^***^ if my doctors refers me to	Mus	Yes (92%)	628 (36)	1126 (64)	0.015
		No (8%)	37 (26)	107 (74)	
	Bahcesehir	Yes (93%)	408 (49)	422 (51)	0.327
		No (7%)	35 (55)	28 (45)	
BC^*^ is the most common cancer in females	Mus	Yes	612 (36)	1092 (64)	0.024
		No	63 (28)	160 (72)	
	Bahcesehir	Yes	352 (52)	329 (48)	0.042
		No	95 (44)	122 (56)	
BC^*^ is curable if detected early	Mus	Yes	632 (36)	1138 (64)	0.016
		No	34 (25)	100 (75)	
	Bahcesehir	Yes	438 (50)	447 (50)	0.786
		No	10 (53)	9 (47)	
MG^***^ exposes to unnecessary radiation	Mus	Yes	365 (32)	771 (68)	0.002
		No	291 (39)	451 (61)	
	Bahcesehir	Yes	80 (38)	129 (62)	<0.001
		No	366 (53)	323 (47)	

## Discussion

BC prevalence is lower, but the proportion of advanced and metastatic BC is higher in underdeveloped and developing countries mostly due to the lack of organized, comprehensive screening programs [10]. The characteristics of the population play a major role in the population’s breast health practices. If the population is carefully studied, screening models can be adjusted, and the success rate of MS programs can be increased [8-9]. With this study, we aimed to compare the MS rate and barriers against MS in the most and the least socioeconomically developed cities in Turkey. We believe our findings will help in designing individualized strategies to increase the success rate of MS both in developing countries, as in Eastern Europe, and also in underdeveloped countries, as in the Middle East.

In the literature, MS rates as high as 80% were reported from socioeconomically high-status populations living in e.g., Scandinavian countries, but the MS rates remain very low in socioeconomically low-status populations [1, 10-14]. Turkey is a developing country, and there are major discrepancies in socioeconomic statuses between west and east regions. The average income in the Eastern Turkey has always been the lowest of all other regions [8]. The gross enrollment rate (GER) of pre-primary education is highest in western regions (19-22%) and lowest in the eastern regions (11%) (Erberber E: Analysing Turkey’s data from TIMSS 2007 to investigate regional disparities in eight-grade science achievement. Erberber E (ed): Unpublished doctoral dissertation, Boston College, Boston, Massachusetts (USA); 2009). According to the Ministry of Development, Istanbul and Mus are socioeconomically the most and the least developed cities in Turkey, respectively [9]. Bahcesehir is a county in Istanbul where homogenously a high socioeconomic status population is living. The MS rate in Bahcesehir was 49% compared to Mus which had an MS rate of 35%. In concordance with the literature, the population with a higher socioeconomic status had a higher MS rate in our study. 

According to the literature, women older than 50 years of age are more likely to adhere to MS guidelines compared to younger women [15]. After the age of 60, this increasing trend turns to a decreasing trend [16]. MS rates were highest in the 50-59 year age group both in Mus (42%) and in Bahcesehir (58%). Also in Bahcesehir the lowest MS rate (37%) was in the 60-69 year age group, probably because less importance is given to health issues after this age. In Mus the lowest MS rate was in the 40-49 year age interval (30%). In Mus 78% of single women were in the 40-49 year age group. In Mus women with no insurance had the lowest MS rate (24%). Especially in rural areas, most of the women are not working as, after getting married, they benefit from their spouse's insurance. Having a health insurance is strongly correlated with participation in MS programs in the literature [10-11, 17-21]. Insured patients have more tendency to perform routine check-ups, which remains the strongest predictor of screening behavior. This might be the reason of lower MS rates among women in 40-49 year age interval and among single women (29%) in Mus. The majority of the women living in Bahcesehir were working and were qualified to have a government insurance on their own. Hence, in Bahcesehir being single was not a barrier against MS, and widowed women had the lowest MS rate (32%). In Bahcesehir women with a monthly income lower than the hunger threshold and Green-Card-holder women had lower MS rates (40% and <1%, respectively). We believe that uninsured women and women with low income should be especially targeted both in socioeconomically low- and high-status populations. We believe expanding the insurance coverage and making MS free of charge would increase the success rate of MS. In socioeconomically high populations, women older than 60 years of age and widowed women; and in socioeconomically low populations, women younger than 50 years of age and single women should be specifically targeted in BC awareness programs.

The most common source of information on BC was audiovisual media (TV & radio) both in Mus (35%) and in Bahcesehir (46%). We believe that audiovisual media should be more effectively utilized both in high and low socioeconomic status populations in increasing the populations' awareness on BC. A physician’s recommendation increases the usage of mammogram significantly [1, 19-21]. MS rate was highest among women who reported doctors as their major information source on BC both in Mus (42%) and in Bahcesehir (67%). In Mus 92% of women and in Bahcesehir 93% of women reported that they would get a mammogram if their doctors referred them to. Women making an annual gynecology visit had a higher MS rate both in Mus (51%) and in Bahcesehir (68%). Women having no breast symptoms had lower MS rates both in Bahcesehir (48%) and in Mus (28%). In Mus MS rates were lower in women with no comorbidities or with a normal body mass index (29% and 27%, respectively). We can speculate that women with no symptoms and no comorbidities visit doctors more seldomly. Our results support that physicians are the most effective information source, and physicians from all specialties should inform their patients on BC and refer women in the risky age group to MS.

The literacy rate was 95% in Bahcesehir and 44% in Mus. The proportion of women who were not an elementary school graduate was 5% in Bahcesehir and 59% in Mus. In the literature, a correlation was reported between educational status, literacy, and BC screening knowledge. According to these studies, low literacy impacts women’s ability to access written cancer-screening materials, benefit from instructions during clinical visits, and apply for health insurance to obtain preventive screening [9, 11, 17]. We believe this major discrepancy in education level is one of the main reasons of the MS rate difference between the two cities. Also in Bahcesehir, women who were non-literate and women who were not graduated from an elementary school had lower MS rates (23% and 23%, respectively) compared to their counterparts. In Mus literate women and women graduated from an elementary school did not have a statistically higher MS rate compared to their counterparts, probably because the proportion of women in the population who were literate and graduated from an elementary school was very low. Women who were daily reading a newspaper had higher MS rates both in Mus (44%) and in Bahcesehir (53%). We believe that non-literate and low-education level women should be specifically targeted while designing BC awareness programs both in high- and low-socioeconomic status populations. As mentioned earlier, audiovisual media is the main information source for BC in developing and underdeveloped countries and should be effectively used to increase awareness.

In the literature, there are studies reporting that women are more likely to adhere to BC screening guidelines when they are more aware and knowledgeable on BC [4-5, 22-24]. In parallel with the literature, women who were aware that BC is the most common cancer in women had higher MS rates both in Mus (36%) and in Bahcesehir (52%). In Mus the MS rate was higher among women who agreed that BC is curable if detected in an early stage (36%). In Mus having a family member with BC increased the MS rate (41%); while in Bahcesehir having a friend with BC increased the MS rate (54%). Women who believed that mammography exposes them to unnecessary radiation had significantly lower MS rates both in Mus (32%) and in Bahcesehir (38%). We believe that to increase the participation rate in MS both in low and high socioeconomic status populations, clear messages should be given frequently on BC being the most common cancer in females, MS enabling early detection and being life-saving, MS being free of charge, and it not causing unnecessary radiation.

This study's limitations were the subjective information gathered via the questionnaires. The population-based model, randomization, high response rates, and large sample sizes were the strengths of our study.

## Conclusions

Low educational level, low-income level, and having no or limited (i.e., Green Card) insurance were the common barriers against MS both in low- and high-socioeconomic status populations. Also women younger than 50 years of age in low socioeconomic populations, and women older than 60 years of age and widowed women in high socioeconomic populations had lower MS rates and should be specifically targeted while customizing BC awareness programs. Lastly, we think that audiovisual media should be used more effectively, and physicians from all specialties should not miss the chance to increase BC awareness both in high and low socioeconomic populations. We believe by keeping these barriers in mind, more successful MS programs can be designed.
